# Book Review: Exploring Language Teacher Efficacy in Japan

**DOI:** 10.3389/fpsyg.2021.812382

**Published:** 2021-12-10

**Authors:** Hongyan Hua

**Affiliations:** School of Foreign Languages, Zhongyuan University of Technology, Zhengzhou, China

**Keywords:** language teacher, efficacy, language teaching, self-efficacy, education

The main objective of “*Exploring Language Teacher Efficacy in Japan*,” written by Gene Thompson, is to provide more food for thoughts for the burgeoning field of language teacher efficacy (LTE) research, by contextualizing it within the Japanese high school language teaching setting. Moreover, this monograph “discusses personal and collective dimensions of language teacher efficacy related to personal second language (L2) capability, instructional L2 efficacy and collective capability toward collaboration” (p. xv). The author conceptualizes self-efficacy as the self-perceptions of teachers about their perceived potentiality to organize and implement courses of action and accomplish desired teaching objectives through both personal and collective efforts. Regarding the intricate, dynamic, and multidimensional interplay between teachers' perceptions, cognitions, affects, and practice, Thompson postulates that affective factors play a crucial role in shaping and determining beliefs that *per se* mediate teacher behaviors in the language classrooms.

Drawing on the sequential mixed-method study, this monograph consists of 11 chapters, each of which looks at self-efficacy for teaching perceptions of Japanese EFL high school teachers. Chapter 1 contextualizes the study in the Japanese language teaching setting, foregrounds the key concepts in LTE, and paves the way for EFL teachers to be confident in using L2 for language instruction. The chapter also emphasizes the challenges to the implementation of Communicative Language Teaching (CLT), highlights the role of English as a medium of instruction (EMI), and pinpoints that not only is teacher self-efficacy is “a type of task-focused confidence” (p. 7), but also “teacher self-efficacy beliefs operate within a theoretical framework” (p. 8) to help L2 teachers and learners move toward confidence.

Chapter 2 delves into the theoretical foundations of teacher efficacy within the theoretical underpinnings of social cognitive theory, by elaborating on the key principles of Rotter's (1966); Bandura's (1977) locus of control and self-efficacy theory, sketching the historical background efficacy beliefs as mediators of emotional, cognitive, and social behaviors, and tracing the development of self-efficacy beliefs. Besides, the chapter brings to the fore a number of avenues for further research by highlighting the interrelationship role between individual and collective efficacy as well as the role of different cultural contexts. More specifically, Chapter 3 concentrates on LTE beliefs, fleshes out the dimensions of LTE, accentuates the role of contextual factors, suggesting that “future studies should explore local factors as potential sources of efficacy information that may contribute to efficacy development or influence perceptions of task difficulty” (p. 43).

Chapter 4 presents the theoretical and approaches to measure language teacher efficacy. The chapter concludes that L2 researchers “(1) establish conceptual clarity of the efficacy construct; (2) locate the research within an appropriate domain of specificity; and (3) develop questionnaire items and interview prompts that operationalise efficacy as future-oriented judgements of capability” (p. 57).

The author cogently argues that one of the concerns for teacher efficacy researchers is how to design questionnaires with robust construct and cultural validity that manifest the real tasks where teacher efficacy perceptions function in specific contexts. To do so, Chapter 5 presents the design process of the efficacy scale utilized in this monograph. Designing the Japanese Teacher of English Teacher Efficacy Scale (JTE-TES), a scale with task and contextual pertinence, is the focus of this chapter. We believe that although this scale has been validated in the Japanese context, it can be valid to scrutinize LTE in other contexts.

Chapter 6 discusses the main findings of the first research question to explore “the underlying dimensions of Japanese high school English teacher efficacy beliefs” (p. 73). Embarking on the semi-structured interviews and exploratory factor analysis with 141 JTEs, it is concluded that some dimensions of LTE have transferable and context-specific features, and there are is contextual influence on some dimensions as well. Chapter 7 scrutinizes the interrelationship between LTE and teacher language proficiency toward the implementation of the L2 in the teaching and learning process. The results corroborate that “Perceived current Eiken level was positively correlated to *Using English, Communicative Teaching, Teamwork and Student Achievement*” (p. 92, emphasis is original). Chapter 8 probes into the L2 instructional efficacy, concluding that “L2 instructional efficacy dimensions are contextually derived” (p. 114). The author reports that communicative teaching and student achievement reflect the two instructional strategy dimensions in the Japanese context.

Chapter 9 focuses on individual, collective, and collaborative teacher efficacy beliefs. It concludes that perceptions about collaborative and collective abilities can potentially impact teachers' efficacy beliefs and their teaching practices. The penultimate chapter concentrates on the construction of LTE cognitions, recapitulating that “experiential activities have a stronger and more predictive relationship as potential sources of teacher efficacy beliefs” (p. 145). Chapter 11 closes the book by highlighting that LTE is a multifaceted, culture-sensitive, and context-specific concept and provides some fresh avenues for future research.

In a nutshell, although set only in the Japanese context, I believe that this monograph has contributed immensely to our understanding of how different personal, social, cultural, emotional, interactional, and contextual factors impact our perceived individual, collective, and collaborative efficacy beliefs. The other merit of the book is to employ sequential mixed methods design to deeply delve into the multidimensional nature of LTE. One interesting finding of this book is the division between self-cognitions of ability about “teaching to encourage communication (*Communicative Teaching*) vs. preparing students for entrance examinations (*Student Achievement*)” (p. 148). So, this monograph is beneficial for language teachers, teacher educators, policymakers, and syllabus designers who consider self-efficacy beliefs as a key and a prerequisite for teachers' continuing professional development.

## Author Contributions

HH has made a direct and intellectual contribution to the work and got it ready for its publication.

## Funding

This study was supported by the Young Backbone Teacher Project of Zhongyuan University of Technology (2019XQG08), the Annual Project of Henan Provincial Social Science Planning Office (2021BYY029), the “Light of Textile” Higher Education Reform Project of China Textile Industry Federation (2021BKJGLX524) and the Henan Provincial Postgraduate Education Quality Project (HNYJS2020KC15).

## Conflict of Interest

The author declares that the research was conducted in the absence of any commercial or financial relationships that could be construed as a potential conflict of interest.

## Publisher's Note

All claims expressed in this article are solely those of the authors and do not necessarily represent those of their affiliated organizations, or those of the publisher, the editors and the reviewers. Any product that may be evaluated in this article, or claim that may be made by its manufacturer, is not guaranteed or endorsed by the publisher.
